# Dynamic Vertical Mapping with Crowdsourced Smartphone Sensor Data

**DOI:** 10.3390/s18020480

**Published:** 2018-02-06

**Authors:** Georgios Pipelidis, Omid Reza Moslehi Rad, Dorota Iwaszczuk, Christian Prehofer, Urs Hugentobler

**Affiliations:** 1 Software and Systems Engineering Research Group, Technical University of Munich, Boltzmannstr. 3, 85748 Garching bei München, Germany; prehofer@fortiss.org; 2 Astronomical and Physical Geodesy, Technical University of Munich, Arcisstr. 21, 80333 Munich, Germany; omidrezamoslehirad2012@gmail.com (O.R.M.R.); dorota.iwaszczuk@tum.de (D.I.); 3 Satellite Geodesy, Technical University of Munich, Arcisstr. 21, 80333 Munich, Germany; urs.hugentobler@tum.de

**Keywords:** indoor mapping, outdoor–indoor transition, CityGML, dynamic mapping, vertical mapping

## Abstract

In this paper, we present our novel approach for the crowdsourced dynamic vertical mapping of buildings. For achieving this, we use the barometric sensor of smartphones to estimate altitude differences and the moment of the outdoor to indoor transition to extract reference pressure. We have identified the outdoor–indoor transition (OITransition) via the fusion of four different sensors. Our approach has been evaluated extensively over a period of 6 months in different humidity, temperature, and cloud-coverage situations, as well as over different hours of the day, and it is found that it can always predict the correct number of floors, while it can approximate the altitude with an average error of 0.5 m.

## 1. Introduction

Indoor maps have become a necessity in robotics, augmented reality, location-based services, mobile ad hoc networks, and search and rescue missions. Because of the high manual effort of generating indoor maps, there have emerged approaches for the dynamic generation of two-dimensional indoor maps through crowdsourced sensor data (e.g., [1,2]). However, these approaches require precise localization. Although many localization providers argue having achieved an average accuracy of 6 m in horizontal localization, none of them provides vertical localization. This has as a result pushed back milestones scheduled by initiatives that are focused on accelerating the research of indoor localization, as these milestones require storey-level localization. Such initiatives are the Enhanced 911 [3] in the United States, and the Enhanced 112 in the European Union [4], as well as the European Accessibility Act [5]. The main reason for the lack of vertical localization providers is the limited information available, for example, the lack of precise altitude indication for every floor in a building in existing maps. To the best of our knowledge, no approach for the dynamic vertical mapping using crowdsourced smartphone sensor data has been proposed.

This paper aims to automate the indoor vertical mapping process, while enriching existing maps with indoor information. In this way, we enable maps to carry information regarding the number of floors in a building and the corresponding altitude of each floor. We achieve this using the novel method we use to fuse the barometric sensor of smartphones with other sensors for the extraction of the ambient reference pressure in locations, which can be used for precise altitude estimation.

More specifically, we first use sensor data extracted from light, proximity, Global Positioning System (GPS), and magnetic sensors to identify the user’s transition from the outside to the inside of a building. Once we recognize this transition, we use it as a landmark for the extraction of the reference pressure. We then use this extracted reference pressure to estimate the altitude differences for every step of the user using the barometric formula. For better clustering between altitude values, we filter out vertical transitions (e.g., stairs or elevators), as they do not belong to floors. Because there is no user who is going to visit all the floors of a building, altitude values from multiple users are aggregated for the identification of the number of floors in a building and the height of each floor. Finally, these data are used to generate three-dimensional (3D) models following the standards as defined by the City Geography Markup Language (CityGML) Level of Detail 2, while an enhancement to the standard models is proposed in order to enable it to carry floor information as well as the altitude of each floor. Various studies attempt to vertically localize humans or objects via pressure sensors [6,7]. However, they all assume reference sensor stations permanently installed in the building. Hence, these are highly infrastructure-dependent approaches. Additionally, several studies attempt to vertically localize objects or humans, mostly triangulating them, using the WiFi received signal strength [8], cellular network antennas [9] or Bluetooth Low Energy (BLE) beacons [10]. Unfortunately, every triangulation method highly depends on the assumption of the existence of particular infrastructure, as well as the line of sight. This means that the strength of the signal, and as a consequence the distance estimation, is influenced when the observer is standing in front of the infrastructure (e.g., BLE beacons) or behind it. Finally, approaches for the dynamic generation of vertical maps have also been proposed [11,12,13]. However, these approaches suggest the use of outdoor characteristics for mapping indoors. This is not feasible, most of the time, as a result of the uniform shape of various buildings, which does not allow any subspace discretization. Additionally, most of the buildings contain underground structures that cannot be recognized through any outdoor model (e.g., subway stations).

Our approach, with an absolute average error of a 0.507 m vertical disposition in three different buildings, although it is infrastructure-independent, performs equally or even outperforms existing approaches, such as in [7], with a 0.8 m vertical disposition, and in [14] with a 0.86 m vertical disposition, which are infrastructure-dependent.

### 1.1. Background on the Barometric Formula

The atmospheric pressure is the weight exerted by the overhead atmosphere on a unit area of a surface. The barometric formula describes how this atmospheric pressure is reduced when the altitude is increased and vice versa. The unit of pressure is 1 hPa = 1 mbar = 100 Pa.

The barometric formula reads:

(1) $$P=P_{b}*{\left[\frac{T_{b}}{T_{b}+L_{b}*\left(h-h_{b}\right)}\right]}^{\frac{g_{0}*M}{R*L_{b}}}$$

where $h_{b}$ is the reference altitude, $T_{b}$ and $P_{b}$ are the temperature and pressure at the reference point, $L_{b}$ is the standard temperature lapse rate of 6.49 K/km, *P* is the pressure at the current point at height *h*, *R* is the universal gas constant 8.3144621 J/K/mol, $g_{0}$ is the earth’s gravity acceleration 9.80665 m/s${}^{2}$ and *M* is the molar mass of the earth’s air 0.0289644 kg/mol.

Equation (1) can be altered for estimating altitude to give the following:

(2) $$h=h_{b}+\frac{T_{b}}{L_{b}}*\left[{\left(\frac{P}{P_{b}}\right)}^{-\frac{R*L_{b}}{g_{0}*M}}-1\right]$$

The barometer equation is valid within a few kilometers of the earth’s surface, within which the lapse rate, gravity acceleration and air composition can be considered constant, given that $P_{b}$ and $T_{b}$ consistently refer to the reference height $h_{b}$. According to the barometric formula, a 1 mbar difference in pressure, with a 15 ${}^{\circ }$C ambient temperature, leads to a 8.33 m altitude change, while a 1 m change of altitude leads to a 0.1201 mbar change in pressure.

### 1.2. Contribution

The contributions of this paper can be summarized as follows:

- We introduce a novel infrastructure-independent method for the dynamic vertical mapping.
- We introduce a novel approach for the reference pressure estimation through the identification of the outdoor–indoor transition (OITransition) of the user through the fusion of three different sensors. In this way, the need of calibration between sensors becomes obsolete.
- We propose an enhancement of the CityGML level of detail two plus (LoD2+) method that provides the indoor geometry of buildings at lower levels of detail.

This paper is an extension of work already presented in [15]. More specifically, in this paper, we have extended the approach, by including an additional sensor for the OITransition discovery. This additional sensor is the magnetic sensor, and more information about it is available in Section 3.3.3. Additionally, the method has been extended and sensor fusion functionality has been added in the reference pressure area component. More information is available in Section 3.3.4. Moreover, the evaluation has been extended with additional collected data over longer period of time, as can be seen in Section 4. Finally, as a result of the above-mentioned extensions of our approach, we have achieved a more accurate identification for the recognition of the OITransition discovery with a true positive score of 99.3% instead of 94.2% in the past. This makes our method more robust against various building characteristics.

### 1.3. Paper Structure

In this paper, the related work is introduced in Section 2; the approach is described in Section 3; the evaluation is presented in Section 4; the paper concludes in Section 5, where limitations to validity are also presented; the resulting models are presented in Appendix A; and the list of collected data is presented in Appendix B.

## 2. Related Work

Enhancing CityGML models with indoor geometry has already been discussed in [11]. In this study, the LoD2+ method was introduced. The method is robust and was implemented successfully using Nef Polyhedra. However, the authors used some prior knowledge, such as building facades and available data modeled following the LoD2 format. As a result, this method is not applicable to general cases because not all buildings contain sufficient information that can be used for mapping indoor areas.

Apple holds a patent that focuses on the visualization of information in indoor 3D places [16]. They do not consider altitude estimation, but instead they assume the existence of indoor maps with locations that specify where vertical transitions may occur, annotated on the map, and a two-dimensional localization mechanism. Additionally, they assume that users can be localized in a particular floor using a particle filter-based framework, which is responsible for assessing the probability of a vertical transition. In this framework, the confidence is quantified on the basis of WiFi access points and the receive signal strength.

Kaiser et al. [17] point out the need of detecting vertical transitions because of the limitation of the Zero Velocity Update (ZUPT) algorithm to identify vertical displacements. To solve this problem, they introduce a moving platform detection module. This works by combining accurate sensors, not those available on a smartphone, such as an accelerometer, barometer and magnetometer. These use ZUPT for localization and a Simultaneous Localization and Mapping (SLAM) algorithm for reducing the remaining drift. They estimate altitude using the barometric sensor, while they also use it to identify landmark phases. In addition, they attempt to identify the boundaries of vertical movements. The intuition for the use of acceleration for the detection of vertical transitions is that the acceleration caused by external factors is weaker than that caused by the pedestrian. However, their approach focuses on correcting real-time localization and assumes the existence of indoor maps.

Li et al. [7] suggest using barometers for 2.5-dimensional (2.5D) (floor-level) localization. They examine how the barometric formula performs for altitude determination. They researched the robustness of altitude estimation on different devices that record differences from 2.1 to 2.5 hPa, which is translated to an offset of multiple floors. They noted that the variation of pressure over 2 h could reach an equivalent of a 10 m height change. They also examined latency robustness as well as stability in the short term, where they noticed changes of 0.1 hPa every 10 min. On the basis of their experiments, they argue that it is impossible to accurately determine height using a barometer in an indoor environment in an absolute manner. They strongly point out the necessity of a reference station. In their study, they used a reference station 5 km away. However, a reference station is not always available, and using other devices such as reference stations requires calibration, which is not realistic in a real-world scenario.

Xia et al. [6] propose the use of multiple barometers as reference points for the floor positioning of smartphones with built-in barometric sensors. This method does not require knowledge of the accurate heights of buildings and storeys. It is robust against temperature and humidity, and it considers the difference in the barometric pressure-change trends and different floors. The intuition is that atmospheric pressure decreases as the altitude increases. Hence, pressure changes that correspond to altitude changes are possible to be calculated using a reference pressure and the barometric formula. As they argue, humidity does not significantly affect the accuracy of the system for indoor altitude estimation; thus, they use the gas constant for dry air and the air molar mass of dry air instead of humid air. On the basis of the barometric formula and using built-in barometric sensors of smartphones as well as information from a local weather station, they are capable of achieving a good discretization between different floor levels. For the current temperature, they consult a local weather station online service. However, this approach is heavily dependent on dense existing infrastructure, while it focuses only on localization and assumes the existence of maps, which describe the location of each sensor.

Bollmeyer et al. [18] use barometers for medical applications in which a precise altitude estimation of the patient’s body is needed. A challenge in this case is the disturbances due to macroscopic flow, such as the influence of ventilation, the opening and closing of doors, or the weather. Calibration between sensors is also needed, in order to compensate for the offset between different sensors. In their research, they created a small sensor network, with sensors attached to the patient body, as well as a reference stationary sensor. They measure a maximum error of 21 cm, but they suggest that a second sensor might reduce the maximum error to 10 cm. However, in our application scenario, we do not focus on such accurate vertical localization; we are looking for an infrastructure-independent approach.

Liu et al. [14] argue that the estimation of altitude via GPS is applicable only outdoors, although even there, its error can be 2.5 times the error of the horizontal location. As a result, they suggest barometers for vertical localization. Their main limitation is the lack of reference points, because the only available reference stations are meteorological stations, which are often sparsely located, while they broadcast periodically, usually at 1 h intervals. Therefore, they introduce the concept of ad hoc reference points. They integrate information from multiple points, while they also use forecast models to estimate air pressure on demand. Besides reference meteorological stations, they additionally use other smartphones when the elevation indication is accurate enough. In order to retrieve better accuracy from other phones, first, they take into account all the reference points that are within a specified distance and time period, and then they give higher weights to reference stations that are closer in distance as well as in time. They also assign a different credibility to different reference stations. Hence, a reference station will be more reliable if its location is known and can report better pressure. They score errors of less than 3 m in outdoor walking, 6 m in mountain climbing, and 0.9 m in indoor floor localization. However, an ad hoc reference sensor reading will constantly have the need of being extracted; it is not clear how this can be achieved, particularly without maps that describe those reference locations.

## 3. Approach

**Figure 1:**
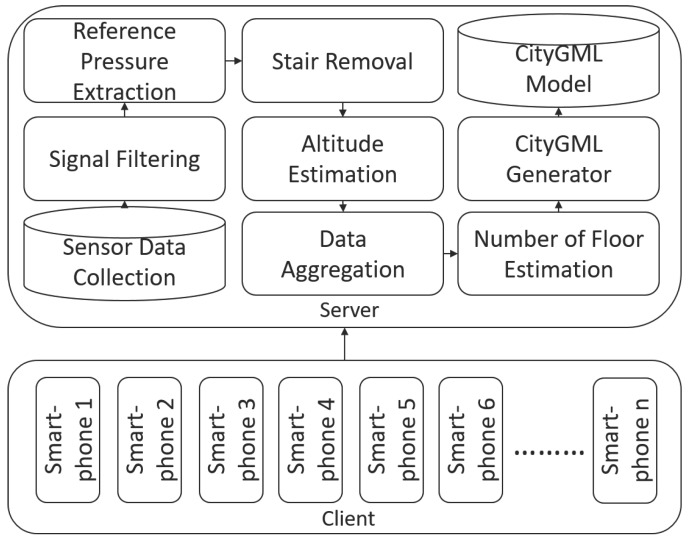
The overall architecture of our system [15].

In this section, we present the main components of our approach. As visualized in Figure 1, the approach is composed of the **Sensor Data Collection** module, which collects the data from smartphone users via an application that has been developed for the purpose of this research and can be found in ref. [19]. After smartphone pressure sensor data are collected, noise is filtered out in the **Signal Filtering** module. The **Reference Pressure Extraction** module has two roles: (1) to filter outdoor data, and (2) to identify locations where pressure readings can be extracted. In the **Stair Removal** module, features that belong to intermediate heights (i.e., stairs or elevators) are rejected. Remaining pressure readings are later used in the barometric formula for **Altitude Estimation**. In the **Data Aggregation** module, we combine data from multiple users, while the **Floor Estimation** module has two roles: (1) to identify the number of floors in a set, and (2) to estimate the altitude of each floor. Finally, in the **CityGML Generator** module, a **CityGML Model** is dynamically generated.

### 3.1. Sensor Data Collection

The sensor data collection module collects sensor data from pressure, light, GPS, proximity and magnetic sensors. Data collected during different temperatures, days, times and humidity situations, labeled with a time-stamp and a unique user identifier, are streamed on a server developed for this purpose through a client–server approach via HTTP protocol, in JSON format. Our collected data are openly available in [20].

### 3.2. Signal Filtering

For smoothing the collected data, the Savitzky–Golay filer [21] is used. Savitzky–Golay is a moving average filter, which applies local regression to a subset of our entire dataset. More specifically, it smooths data by replacing each data point with the average of the neighboring data points within a defined span. This approach is equivalent to

$$y_{s}\left(i\right)=\frac{1}{2N+1}*\left(y(i+N)+y(i+N-1)+\cdots +y(i-N)\right)$$

where $y_{s}\left(i\right)$ is the smoothed value for the *i*th data point, *N* is the number of neighboring data points on either side of $y_{s}\left(i\right)$, and 2N+1 is the span.

### 3.3. Reference Pressure Extraction

The reference pressure is essential for estimating the altitude differences on the basis of the barometer equation using pressure data. The reference pressure is extracted from areas that fulfil the following preconditions: (1) they are common for all user data of each building, (2) they are located indoors, and (3) the pressure fluctuations are low. Such an area is the one that follows the OITransition, as everyone inside a building was at some point in time outside, while it is located indoors where the pressure disturbances are low.

#### 3.3.1. Light Sensor

As has been already suggested by Zhou et al. [22], the OITransition can be identified by aggregating multiple smartphone sensor data. A very promising sensor for this is the ambient light sensor, considering the fact that there is a difference of the light intensity between indoors and outdoors. For identifying the OITransition, in our research, we fuse light and proximity sensors, with 7 and 25 Hz recording rates, respectively. The first sensor helps us to identify the transition, and the second is used as a supportive sensor, indicating when to trust the data, as it can indicate that an object blocks the light sensor.

**Figure 2:**
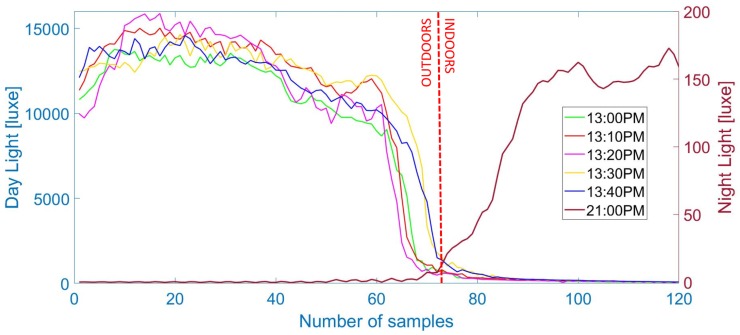
Light data from six outdoor–indoor transitions (OITransitions) collected during the same day, five during day time and one during night. As can be seen, during the OITransition (after the 70th sample), the light intensity rapidly decreases during the day (**left axis**) and increases during the night (**right axis**).

As can be seen in Figure 2, the light intensity drops when entering the building during the day and increases during the night, while the proximity sensor indicates whether to trust the light sensor, because of various phone poses (e.g., phone in pocket). Hysteresis thresholding is used for maximizing the margins of the signal that belong outdoors and indoors. Finally binary classification is applied on the basis of the high and low distribution frequency, while the decision of whether the data is collected during day or night taken from the hour angle $\omega_{0}$ of the sun (negative at sunrise; positive at sunset) is computed with

$$\begin{array}{c} \cos\omega_{0}=-\tan\phi \cdot \tan\delta \end{array}$$

where ϕ is the latitude of the observer on the earth and δ is the sun’s declination.

##### Hysteresis Threshold

**Figure 3:**
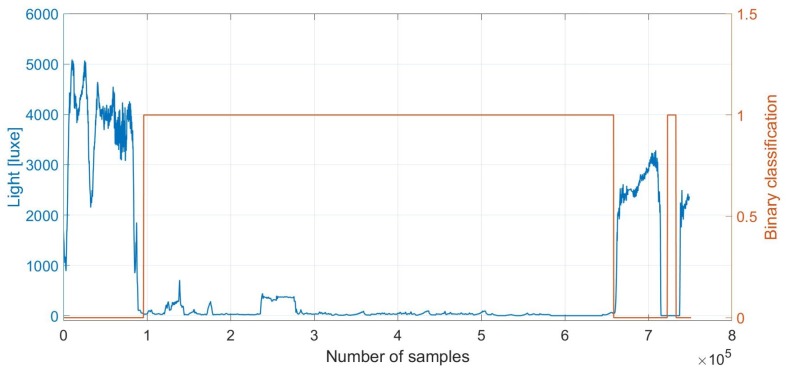
Outdoor–indoor transition (OITransition) classification using light. The binary flag of **1** (orange line and right axis) indicates indoor area. We note that during the period after sample $7\times 10^{5}$, the smartphone was in a pocket. However, it is wrongly classified as indoors. This demonstrates the need for fusion with the proximity sensor, which can indicate whether the phone is exposed (the light sensor can be trusted) or not.

**Figure 4:**
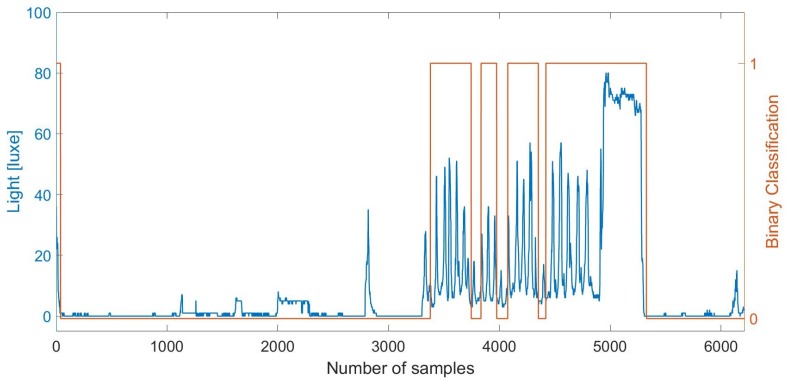
Outdoor–indoor transition (OITransition) classification using light at night. The binary flag of **1** (orange line and right axis) indicates indoor area.

The hysteresis thresholding algorithm uses multiple thresholds to find rapid changes in a signal. The algorithm is thus used to discriminate indoor and outdoor locations. Figure 3 and Figure 4 show that it allows the identification of OITransitions with great accuracy. First, we estimate the upper and lower thresholds for the hysteresis thresholding, on the basis of a histogram analysis. In the histogram analysis, we compute frequency distributions of discrete light intensities. We select the upper and lower thresholds on the basis of the pattern of the distribution. If an OITransition exists in the sensor data segment, then the distribution forms a bimodal pattern and the thresholds are selected from the lower and higher peaks of the distribution. Alternatively, if the sensor data segment contains an OITransition, the distribution shows a symmetric pattern and it is not be taken into consideration as a potential OITransition segment. After the upper and lower thresholds have been defined, the upper threshold is used to find the start of a rapid transition. Once a start point is found, then the path is traced from the rapid signal transition through the signal, segment by segment, marking indoors whenever it is above the lower threshold. It stops marking indoors only when the value falls below the lower threshold.

Unfortunately, as can be seen in Figure 4, the accuracy of the indoor classification at night-time is reduced in comparison to during the day time.

#### 3.3.2. GPS Uncertainty

**Figure 5:**
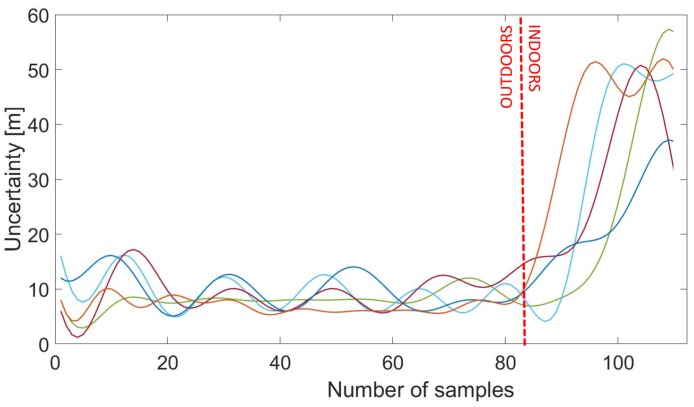
GPS uncertainty data from five outdoor–indoor transitions (OITransitions). As can be seen, at the moment of the transition after the 100th sample, the uncertainty rapidly increased.

**Figure 6:**
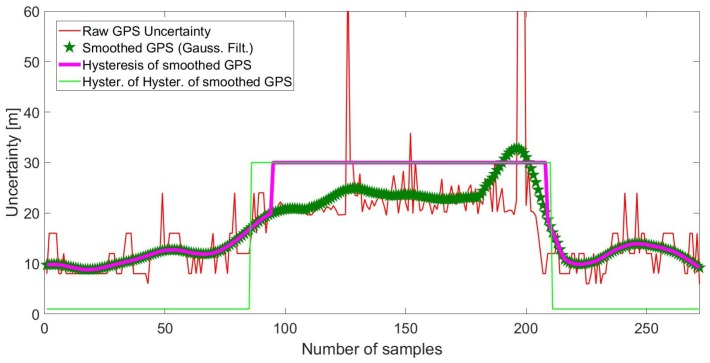
Smoothing and hysteresis thresholding of raw GPS uncertainty signal.

Another characteristic of the OITransition is the rapid increase of the GPS uncertainty. As a result, in our approach, we recorded the GPS uncertainty with a sampling frequency of proximately 1 Hz. As can be seen in Figure 5, at the moment of the transition (after the 80th sample), the GPS uncertainty increased from less than 10 m to almost 60 m. Hysteresis thresholding [23] was applied for the maximization of the margin between low GPS accuracy (indoors) and high-accuracy data (outdoors) for better classification. More specifically, GPS uncertainty was first smoothed via a Gaussian smoothing filter. Then multiple hysteresis thresholding was applied in order to enhance the margin and hence the accuracy of the OITransition classification. The approach can be seen in Figure 6. As can be seen in the figure, raw GPS uncertainty (red line) was first smoothed with a Gaussian filter. Then hysteresis thresholding was applied to the smoothed signal (magenta line).

##### OITransition Detection and Histogram Analysis

Before hysteresis thresholding was applied, the raw GPS uncertainty signal was smoothed via a Gaussian smoothing filter. Then multiple hysteresis thresholding was applied to enhance differences between segments of the signal that belonged outdoors or indoors. This approach is detailed explained in Section 3.3.1 and visualized in Figure 6.

**Figure 7:**
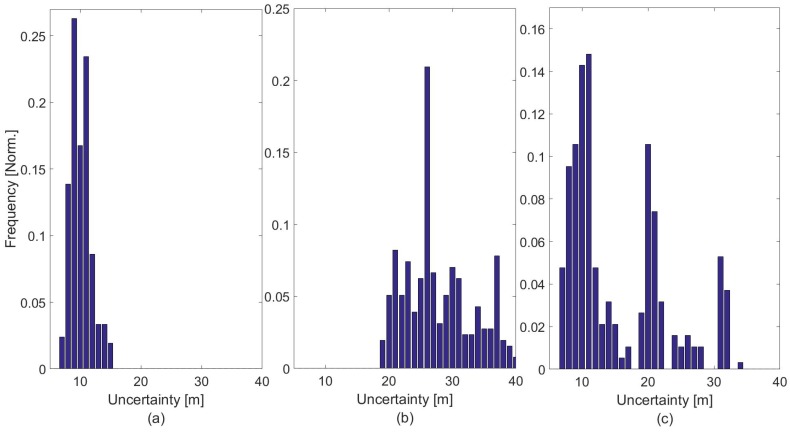
Frequency of GPS uncertainty from data collected from outdoors (a), indoors (b) and during an OITransition.

For the identification of an OITransition in the data segment, as well as for the definition of the threshold in the hysteresis thresholding, histogram analysis was applied in the entire GPS uncertainty signal segment. As can be seen in Figure 7, the frequency of different uncertainty radii is visualized in the histograms. As can be seen in Figure 7c, the histogram forms a bimodal pattern when an OITransition occurs. This is a recognizable characteristic of a segment of uncertainty data that contains an OITransition. Once a transition is identified, the two peaks of the signal are used as the upper and lower thresholds in the hysteresis thresholding algorithm.

On the other side, as can be seen in Figure 7a,b, the histograms show a more symmetric pattern, which is an indication that the data are extracted from a single place; this place is either indoors or outdoors. More specifically, as can be seen in Figure 7b, the GPS uncertainty is high—more than 20 m—which is an indication that the particular segment has been extracted from exclusively indoor locations. On the other side, as can be seen in Figure 7a, the GPS scores a low uncertainty—less than 15 m—which is an indication that the data are extracted from exclusively outdoor locations.

#### 3.3.3. Magnetic Signal

**Figure 8:**
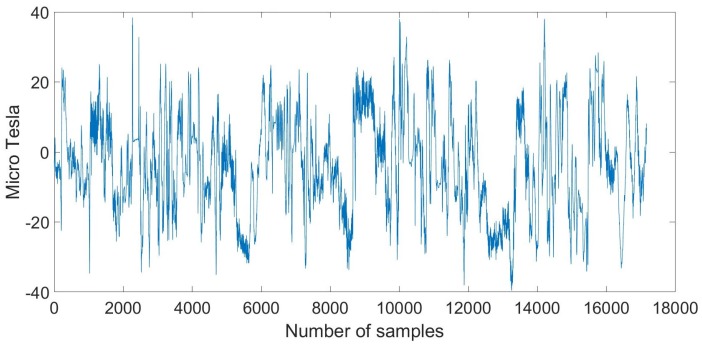
Magnetometer signal from walking into four consecutive buildings.

The magnetic sensor can detect disturbances of the ambient magnetic field, as a result of steel elements inside the walls of a building. Hence, the intensity of the magnetic field can be used as an indicator for identifying the OITransition [24]. In this section, we introduce a process for the identification of the OITransition by measuring the disturbances of the magnetic field. For the identification of OITransitions, we combine a Gaussian filter and the moving window standard deviation. In the following example, we selected a magnetic dataset from the collected data [20] from four of our buildings. The corresponding magnetic signal is shown in Figure 8:

The route that corresponds to the signal shown in Figure 8 begins outdoors, followed by four indoor transitions and four outdoor transitions. Towards the end of the time interval, the third outdoor transition occurred when exiting the fourth building.

**Figure 9:**
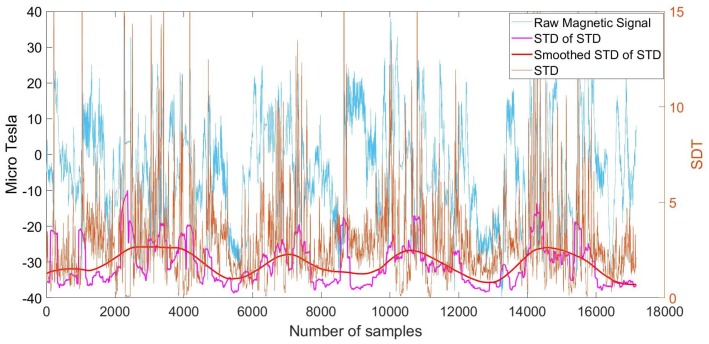
Magnetometer signal from walking into four consecutive buildings and corresponding smoothed moving Standard Deviation (STD) of moving STD with kernel size of 500.

In the first step, disturbances in the signal were found using the moving window standard deviation with a window size of 20 samples along the time axis. The resulting signal is shown in Figure 9 (orange line). Once disturbances were identified, a second moving standard deviation extraction was applied to the new generated signal. This time, the window size corresponded to 200 samples. The result is illustrated in Figure 9 (purple line).

In the third step, a Gaussian filter was applied to the resulting signal in order to smooth it with a kernel of 500 samples. As can be seen in Figure 9 (red line), this contributed to the identification of the four blobs that correspond to the duration—one by one—of the indoor walking activities. They then could be used to distinguish indoors from outdoors.

**Figure 10:**
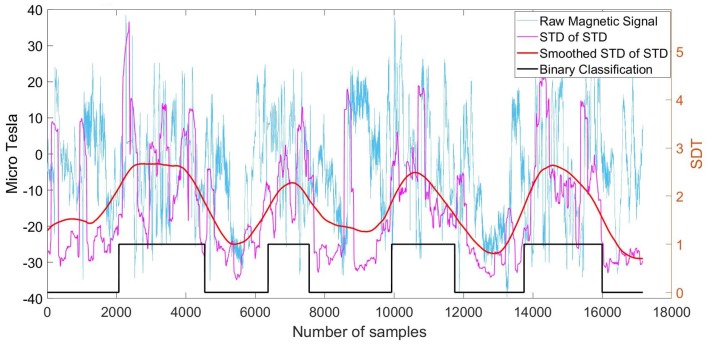
Magnetometer signal from walking into four consecutive buildings and corresponding smoothed moving STD of the disturbance, with kernel size of 200 samples, and the final binary classification.

Finally, in order to enable binary classification between indoor and outdoor areas, a moving STD was performed, followed by another Gaussian filtering step. The resulting signal was then used to determine the start and end of the indoor areas. The final classification can be seen in Figure 10 (black line), where the value 1 corresponds to indoors and 0 corresponds to outdoors.

#### 3.3.4. Fusion

The sensor fusion was made as is described in Table 1. The sensors that have been taken into consideration are the proximity, the light, GPS and the magnetic field sensor. Their decision is fused as follows:

- If the proximity sensor indication is false, this implies that there is no obstacle blocking the light sensor. As a result, three sensors are available. Hence, the result is determined on the basis of the voting fusion. For example, if the light and GPS sensors identify that the particular data segment is extracted from indoors, then the segment is classified as an indoor data segment.
- On the other side, if the proximity sensor indicates “true”, then we have only two sensors available. The majority voting can thus not be applied here. Hence, in such a case, the logic operation and is applied. For example, if the magnetic sensor indicates disturbances—and as a result, indoors—but the GPS uncertainty is low, which indicates outdoor space, then the segment is classified as outdoors.

### 3.4. Stair Removal

In the stair removal phase, sets of features with high disturbances in the pressure readings are rejected, as they mostly correspond either to vertical transitions (e.g., stairs or elevators) or to outliers (e.g., high wind velocities). Such features of high disturbance are identified using the moving window standard deviation.

This approach is equivalent to

$$\sigma =\sqrt{\kappa }$$

where

$$\kappa =\sigma^{2}=\frac{1}{N-1}\left(q-\frac{s^{2}}{N}\right)$$

with

$$q=\sum_{i=1}^{N}x_{i}^{2}\;\mathrm{and}\;s=\sum_{i=1}^{N}x_{i}$$

where $x_{i}$ is the instance of the input signal and *N* is the number of elements.

### 3.5. Altitude Estimation

The altitude is estimated on the basis of the barometer Equation (2) as follows:

(3) $$h=\left[{\left(\frac{P_{0}}{P_{i}}\right)}^{\frac{1}{5.25}}-1\right]*\frac{T_{b}+273.15}{0.0069}$$

where $P_{0}$ is the reference pressure extracted from the location where the OITransition was identified, $P_{i}$ is the current pressure value and $T_{b}$ is the temperature value in °C, which is extracted via openly available weather stations online.

### 3.6. Data Aggregation

Data aggregation is essential for identifying all floors inside a building, as not all users are expected to visit all floors. In the data aggregation module, multiple recorded data are fused. Grouped by their GPS coordinates and combined with the building outline, extracted from OpenStreetMap [25], it is ensured that the data always correspond to the same building. More specifically, altitude information estimated from multiple users and labeled by their unique users identifier (UUID) are sorted by their time-stamp and fused together for the classification phase. Because the reference pressure for the altitude estimation is extracted by the same device as that used for estimating it, approximately at the same location for all the users, because of the novel approach for reference altitude extraction on the basis of the identification of the OITransition, there is no need to calibrate any sensor between different phones. In this paper we consider all existing entrances of a building to be at the same altitude. However, in the case of multiple entrances at different altitudes, the entrance altitude, as well as the longitude and latitude, can be extracted from [25], and then the OITransition can be used for the identification of the entrance location. Once the entrance location is identified, the difference between the global altitude of the entrance can be used for locally referring the floor height.

### 3.7. Number of Floors Estimation

Because the number of floors as well as the label of every floor (i.e., the corresponding altitude) are unknown, for classification, we used a classifier able to cope with unlabeled data. The classifier K-means was selected because of its simplicity and its relatively low processing demand. For estimating *K*, the elbow method was selected. The classification process is divided into two main steps. The first step is the identification of *K*, which corresponds to the true number of floors. In the second step, the center of each cluster is recognized, which corresponds to the altitude of every floor.

#### 3.7.1. Identification of *K*

Because the number of floors is unknown (*K*), it has to be estimated in the first step. For this purpose, the elbow method [26] was chosen. The elbow method is a clustering analysis method, and it enables the interpretation and validation of the consistency within the cluster analysis. It takes into consideration the percentage of variance explained as a function of the number of clusters: the optimum number of clusters is reached when adding another cluster no longer improves the modeling of the data. If we plot the variance as a function of the number of clusters, the first clusters will add much information, but with an increasing number of clusters, the marginal gain will drop and the graph will flatten out, indicating the optimum number of clusters. Identifying the correct number for *K* is essential, as it corresponds to the number of floors. A wrong estimation of *K* can lead to large errors in the estimated altitude of each floor.

#### 3.7.2. The Centroid of the Clusters

After *K* is identified, the classification is made using K-means, as the cluster label (i.e., the altitude of each floor) is unknown. The input to the algorithm is the computed vector of filtered pressure data and the estimated number of floors. The algorithm’s output is then a vector with the assigned classes for every input point and the cluster centroids.

### 3.8. Implementation in CityGML

**Figure 11:**
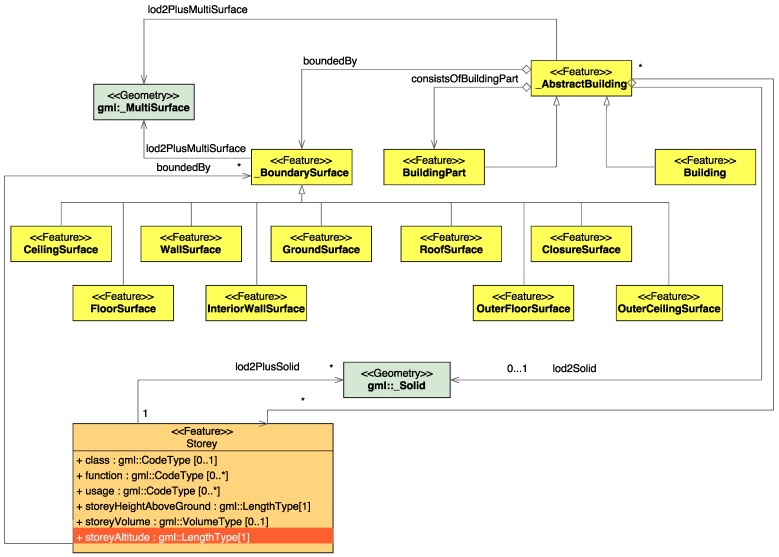
The proposed level of detail two plus (LoD2+) model, which carries information about the number of stores as proposed by [11] and their corresponding altitudes.

In our research, we concentrate on the derivation of the floor numbers and their heights. This does not allow us to create a complete LoD4 model. As a result, we enhance the LoD2 model geometry with the hull geometry for each floor. For this purpose, we introduce LoD2+, as visualized in Figure 11.

In LoD2 and higher LoDs, the outer facade of a building can be modeled semantically by the _BoundarySurface. The _BoundarySurface is a part of the building’s exterior shell with an assigned function such as the wall WallSurface, roof RoofSurface, ground plate GroundSurface, outer floor OuterFloorSurface, outer ceiling OuterCeilingSurface or ClosureSurface. For indoor modeling FloorSurface, InteriorWallSurface, and CeilingSurface can be used [27]. In [11], the authors enhance the CityGML scheme with a new feature class, Storey, which has five attributes: class, function, usage, storeyHeightAboveGround and storeyVolume.

To model the indoor geometry, we keep the LoD2 representation using _BoundarySurface and add indoor geometry for each storey using FloorSurface, InteriorWallSurface, and CeilingSurface, as well as the feature class Storey introduced by [11]. In addition, we propose a further attribute of the feature class Storey: storeyAltitude. This attribute is necessary for our application, as the output of a navigation device is an altitude and not the height above the ground. This extension is not included in the current version of the CityGML specification, however we suggest to include it in the next release.

For the dynamic generation of the CityGML model, citygml4j [28] was used. This is an open-source library for Java, which binds the XML Schema definitions of CityGML to a Java object model.

## 4. Evaluation

In this section, we present the evaluation of the proposed method for the dynamic vertical mapping from user smartphone data as shown in Table 2. More specifically, in Section 4.1, the difference in calibration between the two phones used in this experiment is presented. Section 4.2 presents the robustness of our algorithm against various human walking velocities. In Section 4.3, the performance of the identification of OITransitions is evaluated. In Section 4.4, the evaluation of the identification of the number of floors and their altitude estimation during various weather conditions is presented. For the evaluation of the stair removal Section 4.2, data were collected from three different human walking velocities. Finally, a detailed evaluation, with datasets collected over a period of 6 months from three different buildings, is presented in Section 4.4.

### 4.1. Different Phone Calibration

**Figure 12:**
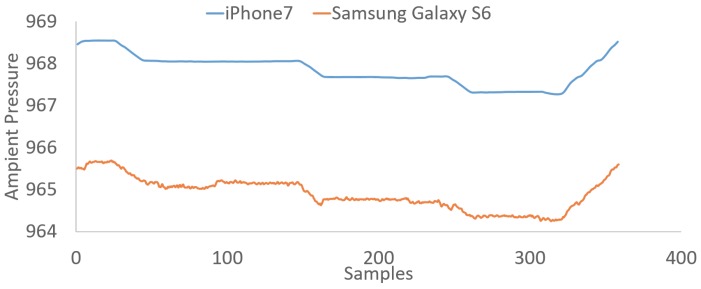
Data collected from an iPhone 7 and a Samsung Galaxy S6, while the user had climbed three floors upwards and the same number of floors downwards.

In this section, we discuss the use of our algorithm for two different smartphones. As can be seen in Figure 12, there is an offset between the sensor readings of the two phones. This implies that there cannot be a single point of reference for both sensors and highlights the need for calibration between the two phones. However, as can be seen, the offset between the two sensors is almost stable. As a result, this effect demonstrates the need of self-reference that our approach offers. Hence, considering the fact that each phone will extract reference pressure from its own sensor and the fact that the offset between different phones is stable, our proposed approach will work for any given barometric sensor calibrated under any given circumstances.

### 4.2. Evaluation of Stair Removal

**Figure 13:**
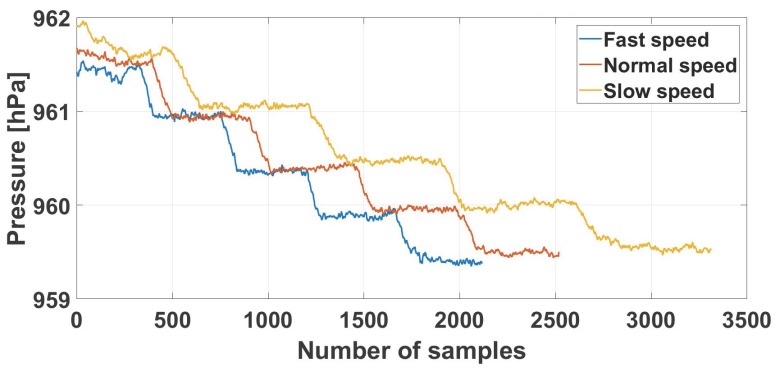
Dataset used for the evaluation of the stair removal method. The data was collected from the same route for three different visits and walking velocities, approximately 1×, 1.5× and 2× [15].

For testing the robustness of our algorithm against different walking velocities in the stair removal component, we recorded data with three different walking velocities, approximately 1×, 1.2× and 1.5×, while climbing five pairs of stairs on a building, as can be seen in Figure 13. As demonstrated in the results (Table 3), the algorithm scored a precision of 94%, recall of 93.8% and F-score of 93.9% on correctly identifying the stairs, with the same sliding window length for all datasets. The sliding window size was 50 samples long or approximately 10 s, while it slid for every sample or approximately every 250 ms.

### 4.3. Evaluation of Reference Pressure Extraction

The reference pressure value for the altitude estimation with the barometric formula corresponds to the location that follows the entrance of a building, as detailed described in Section 4.3. As a result, the identification of the OITransition is necessary in order to identify the building entrance. The transition is identified by monitoring peaks and drops by monitoring peaks and drops in the readings of a number of sensors and their fusion, as suggested by [22] and described in Section 4.3. However, in our scenario, the ambient light, the GPS uncertainty and the disturbances of the magnetic field are taken into consideration, rather than the WiFi Received Signal Strength (RSS) and the Global System for Mobile Communications (GSM) RSS. The approach has been evaluated in three different buildings with four collected datasets for each building, during day and night. Our collected data and the algorithm used for the evaluation are open-source and can be found in [20].

**Figure 14:**
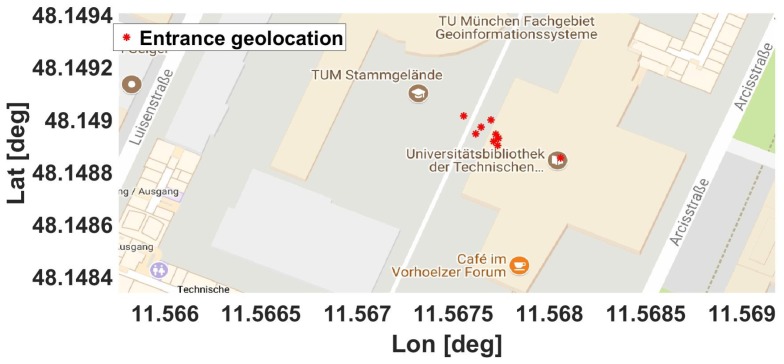
Locations that correspond to the detection of the outdoor–indoor transition (OITransition). The figure includes nine different determined locations for the entrance to the building (red dots) [15].

As can be seen in Figure 14, the OITransition (red dots) was successfully identified in all of our datasets. Additionally, the entrance location could also be approximately determined by our approach. This was considered as the place of the transition, for example, the space between the last low GPS uncertainty values and the first high GPS uncertainty values. As a result, we could additionally estimate the spatial error of our approach for the OITransition identification. Hence, the entrance location latitude has been approximated by an average of 1.6 m, while the entrance longitude has been approximated by an average of 5.5 m. This score was lower than the GPS average error outdoors, which was between 10 and 12 m.

Furthermore, five out of nine times, the entrance location was identified at the latitude of 48.1489, while two times it was identified at the latitude of 48.14895 and once it was identified at the latitudes of 48.14885 and 48.149. The final latitude was to be decided on the basis of the median, which was 48.14894251, while the true entrance latitude as mapped in the open street maps was 48.1489277. Hence, our algorithm scored an error of 0.00001${}^{\circ }$, which corresponded to less than 1.64 m. Regarding the longitude, three out of nine times the entrance was localized at the longitude 11.5677, twice at 11.56775 and once at 11.56755, 11.5676, 11.56765 and 11.568. The final entrance location was estimated from the median at 11.568, when the true entrance was located at longitude 11.568. As a result, our algorithm had an error of 0.00004${}^{\circ }$, which corresponded to approximately 4.614 m.

Finally, in Table 4, Table 5 and Table 6, a detailed evaluation of the OITransition determination for each sensor and the sensor fusion for all 3 buildings and 12 datasets is presented. According to the tables, our algorithm scored an average of 96.8% for precision, 94.2% for recall and 95.5% for the F-score, for identifying the OITransition using a GPS sensor. It scored 93.6% for precision, 96.3% for recall and 94.9% for the F-score for OITransition detection with a light sensor. It scored 88.8% for precision, 89.2% for recall and 89% for the F-score for OITransition detection with a magnetic sensor. It scored 99.4% for precision, 90.7% for recall and 94.8% for the F-score for the fusion of all sensors on the basis of the voting fusion. When the light sensor was not available or when the proximity sensor indication was true, it scored 99.1% for precision, 97.3% for recall and 98.2% for the F-score.

As a result, we can conclude that the OITransition can be recognized and represents a robust means for the extraction of the reference pressure. Additionally, the GPS sensor scored the lowest false positives, while the light sensor scored the lowest false negatives. Furthermore, the fusion of the three sensors scored the lowest false positive rate, and the false positive rate dropped only by 0.3% when the light and magnetic field sensor were the only sensors that were fused.

### 4.4. Evaluation

This section presents a long-term evaluation for the number of floors and the floor heights determined for the buildings TUM main campus (library; Section 4.4.1), Building B (Section 4.4.2), and Deutsche Akademie (Section 4.4.3). The ground truth was obtained via a high-precision laser range meter device. We observed these buildings for about 6 months to evaluate the effects of long-term weather conditions on the measurements.

#### 4.4.1. Building 1: TUM Main Campus

TUM main campus has five floors and a ground floor. The true height for each floor is listed in Table 7. Nineteen datasets were collected from TUM main campus, over a 4 month period. The average duration of the datasets collected was 14.2 min, with an average of 3204 samples from the pressure sensor. All the collected datasets are available in [20]. We collected data from various hours during daylight and night; different routes were traveled inside the building, at different temperatures, humidity levels and ambient pressure, and finally with different cloud coverage. After smoothing and clustering the data as explained in Section 3.2, the OITransition was identified as described in Section 3.3. The accuracy of this component is presented in Section 4.3, in Table 4. Once the OITransition was estimated and the reference pressure was extracted, the altitude of every pressure reading that belonged to indoors was computed. Once all the pressure readings were translated into altitude, they were imported to the elbow method for floor number identification.

**Figure 15:**
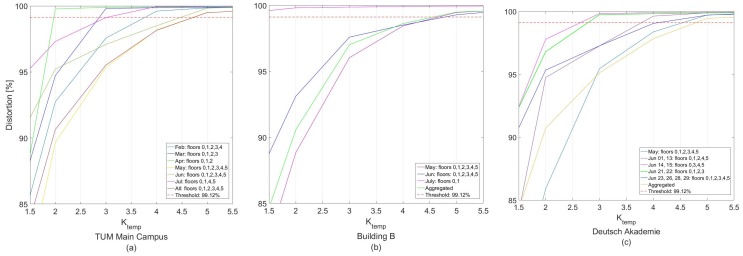
Elbow method result for three test buildings (**a**) TUM Main Campus, (**b**) Building B, (**c**) Deutsche Akademie.

As can be seen in the elbow method results, in Figure 15a, the number of floors (i.e., clusters, *K* = 6) in our dataset has been identified correctly for the aggregated dataset as well as for the May and June datasets, for which all floors of the building were visited. The threshold selected for all datasets was 99.12% for the distortion percentage, and for the clustering, the K-means algorithm was selected. Additionally, it can be seen that for the February dataset, the number of floors predicted was five ($K_{temp}=4$), as the fifth floor was not visited during this month. For March, the predicted number of floors was four ($K_{temp}=3$), as the two highest floors were not visited during that month. For April, the predicted number of floors was three ($K_{temp}=2$). Finlay, in the datasets extracted during July, the predicted number of floors was four ($K_{temp}=3$), and the third cluster’s distortion fell slightly above the 99.12% threshold.

**Figure 16:**
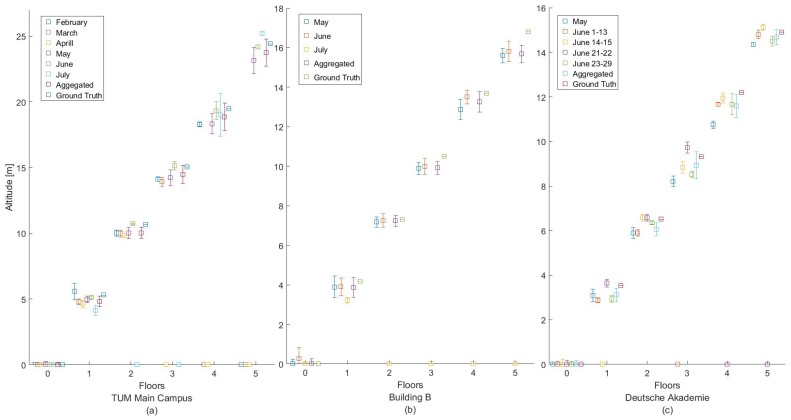
Estimated altitude and ground truth for each floor height for three test buildings (**a**) TUM Main Campus, (**b**) Building B, (**c**) Deutsche Akademie.

To demonstrate the performance of the altitude estimation or the label of each class (i.e., the centroid of each cluster), the corresponding estimated floor altitude is visualized together with the ground truth in Figure 16a and is listed in Table 7. As can be seen for the aggregated dataset, the maximum error was at 0.66 m, while the minimum error was at 0.48 m. In Figure 16a, it can also be seen that the fact that some datasets were non-visited floors (i.e., July, April, March and February) did not cause a problem to our database, as these floor altitudes were ignored.

#### 4.4.2. Building 2: Building B

Building B consisted of five floors and an additional ground floor. The true height for each floor is available in Table 8. We have collected data following the same strategy as mentioned above. Twenty-five datasets were collected from Building B in Munich. All collected datasets are available in [20]. After following the procedure described above, for smoothing, clustering and identifying the OITransition, we extracted the reference pressure and then the altitude of every pressure reading that belonged indoors. Finally, once we translated all the pressure readings into altitudes, they were imported to the elbow method for floor number identification.

As can be seen in the elbow method results, in Figure 15b, the number of floors (i.e., clusters, *K* = 6) in our dataset has been identified correctly for the aggregated dataset as well as for the May and June datasets, for which all floors of the building were visited. The threshold selected for all datasets was 99.12% for the distortion percentage, and for the clustering algorithm, the K-means algorithm was selected. Additionally, it can be seen that for the July dataset, the number of floors predicted was two ($K_{temp}=1$), as the four higher floors were not visited during this month.

Regarding the height estimation, the corresponding estimated floor altitude and ground truth are presented together in Figure 16c, as well as in Table 8. As can be seen, in the aggregated dataset, the maximum error was at 1.12 m, while the minimum error was at 0.31 m. In the figure, it can also be seen that for the July dataset, only two floors were visited.

#### 4.4.3. Building 3: Deutsche Akademie

We collected 20 datasets from Deutsch Akademie. This consists of five floors and a ground floor. All collected datasets are available in [20]. The true height for each floor is available in Table 9. Once we estimated the OITransition and extracted the reference pressure, then the altitude of every pressure reading that belonged indoors was computed. Once all the pressure readings were translated into altitude, they were imported to the elbow method for floor number identification.

The ground truth of this building is illustrated in Table 9.

As can be seen in the elbow method results, in Figure 15c, the number of floors (i.e., clusters, *K* = 6) in our dataset has been identified correctly for the aggregated as well as the May and June datasets, for which all floors of the building were visited. The threshold selected for all datasets was 99.12% for the distortion percentage and the clustering algorithm was selected for the K-means algorithm. Additionally, it can be seen that for the June 1 and 13 datasets, the number of floors predicted was five ($K_{temp}=4$), as the third floor was not visited during this period. For June 14 and 15 as well as for June 21 and 22, the predicted number of floors was four ($K_{temp}=3$), as the two floors were not visited during these period. More specifically, the non-visited floors were the first and second, for the first dataset and the two highest floors for the later dataset.

On the other hand, the corresponding estimated floor altitude and ground truth are visualized together in Figure 16c and Table 9. As can be seen for the aggregated dataset, the maximum error was at 0.61 m, while the minimum error was at 0.23 m. In the figure, it can also be seen that some datasets being non-visited floors (i.e., June 14–15 and 23–29) did not cause a problem to our database, as these floor altitudes were ignored.

## 5. Conclusions

This paper describes our novel framework for the dynamic mapping of the vertical characteristics of a building. The proposed method makes use of a new sensor available in the latest smartphones (the last from 2017), and the barometric sensor, which indicates the ambient pressure and manages uncertain sensor data collected from crowdsourcing. The method estimates the altitude of the collected data with the use of the barometric formula. For achieving this, we introduce a novel approach for the extraction of the reference pressure at the OITransition of the user, which is identified through sensor fusion. More specifically, the GPS uncertainty, the magnetic disturbances and the ambient light are taken into consideration for identifying the transition, while the proximity sensor is also used as a supportive sensor. We faced an unsupervised classification problem, in which the number of floors—or the number of clusters—as well as the altitude—or the label of each class—for each floor were unknown. To resolve this problem, a clustering analysis technique called the elbow method and the popular K-means clustering algorithm were used. Finally, we propose a way to map these characteristics by enhancing the standards of the CityGML, enabling it to carry information about the vertical characteristics of a building in lower LoDs.

Although it has been demonstrated in the paper that our approach can work with any barometric sensor (Section 4.1), as the offset between different barometric sensors is stable, our approach has been extensively evaluated only for the Samsung Galaxy S6 [29].

Additionally, we noticed that when a significant delay follows the OITrsansition and precedes ascending to different floors, the vertical localization error increases. This is due to the long-term instability of the ambient air pressure. The same happens when there is lack of data from one floor. It is very likely that such data will not be taken into consideration in the clustering analysis and finally in the clustering phase. This will result in a missing floor in the final model.

## Figures and Tables

**Table 1:** Fusion Rules.

Proximity	Light	GPS	Magnetic	Indoor	Outdoor	Fusion Model
False	0	0	0	F	T	Voting
False	0	0	1	F	T	Voting
False	0	1	0	F	T	Voting
False	0	1	1	T	F	Voting
False	1	0	0	F	T	Voting
False	1	0	1	T	F	Voting
False	1	1	0	T	F	Voting
False	1	1	1	T	F	Voting
True	—	0	0	F	T	and
True	—	0	1	F	T	and
True	—	1	0	F	T	and
True	—	1	1	T	F	and

**Table 2:** Collected Data used for evaluation. The table shows the date of collecting the data, the time, the indicated temperature from AccuWeather (T A) and Google (T A) (unit: °C), the relative humidity from the same two sources (H A) and (H G), and the ambient pressure from AccuWeather (P A) (unit: Pa). The buildings belong to the Technical University of Munich (TUM) main campus area and are (1) Agness 27, (2) Adelheid 13A, (3) Agness 33 and (4) TUM main campus.

Date & Time	T A	T G	H A	H B	P A	ID
May 10, 10:20	9	10	70	74	1011	1
May 10, 21:40	11	13	61	45	1006	1
May 12, 18:20	21	19	40	52	1004	1
May 9, 17:00	10	9	49	52	1016	1
May 9, 10:40	8	9	75	72	1017	2
May 9, 17:30	10	11	49	55	1016	2
May 10, 22:00	11	11	61	65	1006	2
May 12, 18:30	21	19	40	45	1004	2
May 9, 10:10	8	9	75	60	1017	3
May 9, 16:40	10	9	49	59	1016	3
May 10, 10:00	9	8	70	40	1011	3
May 12, 17:50	21	19	40	43	1004	3
Feb 11, 14:30	6	2	70	72	1019	3
Feb 12, 19:00	0	1	87	80	1028	3
Feb 21, 21:30	7	0	93	83	1017	3
Mar 21, 13:30	13	8	58	64	1010	3

**Table 3:** Confusion matrix of stair removal.

	Fast		Normal		Slow	
	Floors	Stairs	Floors	Stairs	Floors	Stairs
Floors	1584	58	2037	0	2683	0
Stairs	179	296	76	404	157	472

**Table 4:** Confusion matrix of Building I.

	GPS		Light		Magnetism		Fusion	
	Indoor	Outdoor	Indoor	Outdoor	Indoor	Outdoor	Indoor	Outdoor
Indoor	614	21	6121	210	3323	519	1,162,298	302,269
Outdoor	21	696	163	5526	144	2776	1329	746,993

**Table 5:** Confusion matrix of Building II.

	GPS		Light		Magnetism		Fusion	
	Indoor	Outdoor	Indoor	Outdoor	Indoor	Outdoor	Indoor	Outdoor
Indoor	390	3	5748	1069	2911	460	1,228,507	25,114
Outdoor	20	805	220	4428	0	2383	6915	820,470

**Table 6:** Confusion matrix of Building III.

	GPS		Light		Magnetism		Fusion	
	Indoor	Outdoor	Indoor	Outdoor	Indoor	Outdoor	Indoor	Outdoor
Indoor	127	0	4963	154	7749	179	1,186,784	41,546
Outdoor	29	184	264	3788	1552	3571	13,700	924,258

**Table 7:** Ground truth, estimated altitude and error for Technical University of Munich (TUM) Main Campus.

Floors	0	1	2	3	4	5
Real floor altitude (m)	0	5.3	10.68	15.05	19.47	24.41
Estimated floor altitude (m)	0	4.81	10.03	14.48	18.86	23.74
Error	0	0.48	0.65	0.57	0.61	0.66

**Table 8:** Ground truth, estimated altitude and error for Building B.

Floors	0	1	2	3	4	5
Real floor altitude (m)	0	4.17	7.31	10.5	13.7	16.8
Estimated floor altitude (m)	0.022	3.86	7.24	9.92	13.26	15.68
Error	0	0.31	0.073	0.585	0.44	1.12

**Table 9:** Ground truth, estimated altitude and error for DeutschAkademie.

Floors	0	1	2	3	4	5
Real floor altitude (m)	0	3.54	6.51	9.31	12.2	14.9
Estimated floor altitude (m)	0	3.1	6	8.9	11.59	14.67
Error	0	0.4	0.45	0.38	0.61	0.23

**Figure A1:**
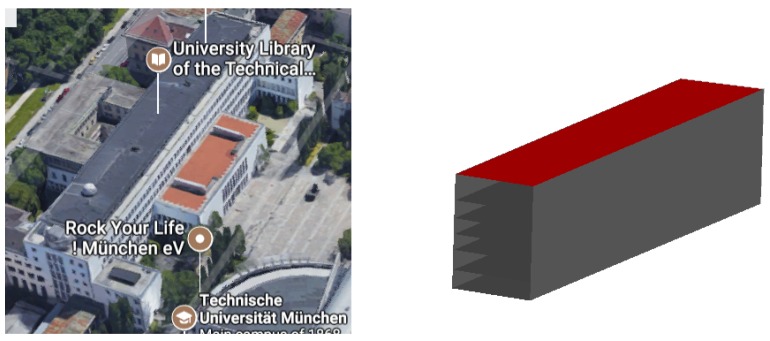
Final CityGML level of detail two plus (LoD2+) model for the TUM main campus building.

**Figure A2:**
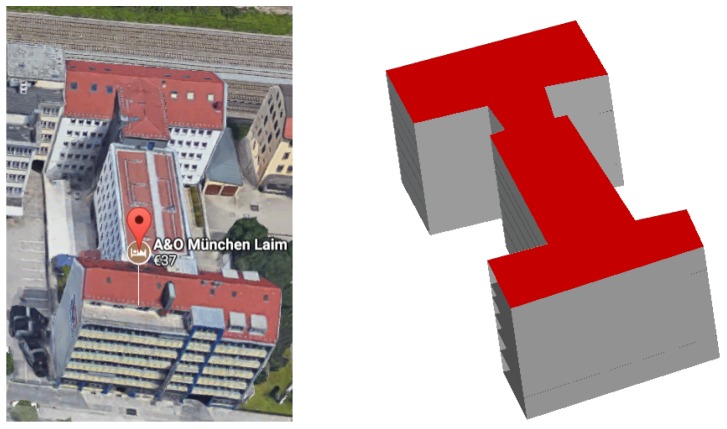
Final CityGML level of detail two plus (LoD2+) model for Building B.

**Figure A3:**
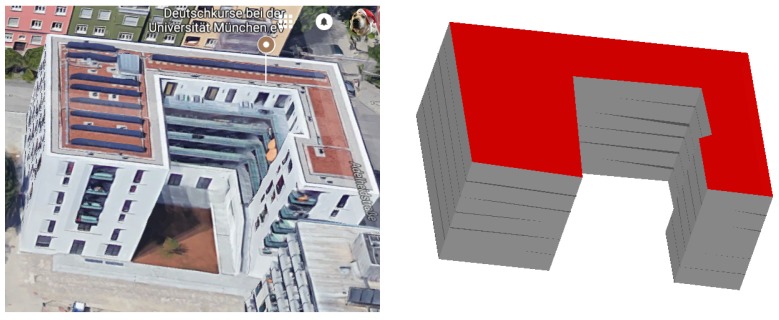
Final CityGML level of detail two plus (LoD2+) model for the Deutsche Akademie building.

## Appendix A. Appendix I: Final Models

In this section, the final models together with photographs of the buildings are presented. Those models have been generated dynamically and without the intervention of a user. The outline of the buildings has been extracted from [25]. The CityGML models are available in [20].

## Appendix B. Appendix II: Collected Data

In this section, we list all the data that were collected for the evaluation of our model. All the collected data are available in [20], and they include measurements from the following sensors: acceleration, gyroscope, pressure, light, proximity, GPS, magnetometer, pedometer, WiFi, pressure and GSM sensors.

**Table A1:** Collected data from TUM main campus. The table shows the data acquisition date, the time, the visited floors (V Floors), the indicated temperature from AccuWeather (T A) and Google (T A) (unit: °C), the relative humidity from the same two sources (H A) and (H G), and the ambient pressure from AccuWeather (P A) (unit: Pa).

Date	Time	V Floors	T A	T WC	H A	H WC	P A	P WC	W S	Cloud Cov.
10 Feb	4:30 PM	0, 1	−1	−2	91%	82%	1020	1020	Normal	NG
11 Feb	3:30 PM	0, 1, 3, 4	6	6	87%	84%	1020	1021	Normal	NG
12 Feb	8:00 PM	0, 2	0	1	87%	79%	1028	1027	Normal	NG
27 Feb	4:00 PM	0, 1	14	13	38%	70%	1014	1014	Normal	13%
18 Mar	8:30 PM	0, 3	9	8	81%	64%	1010	1011	Normal	100%
21 Mar	2:30 PM	0, 1, 2	13	13	58%	56%	1010	1010	Normal	90%
26 Mar	12:30 AM	0, 1, 2, 3	4	2	80%	62%	1020	1023	Normal	0%
28 Mar	10:00 AM	0, 1	6	8	60%	69%	1024	1024	Normal	0%
7 Apr	09:30 PM	0, 1, 2	11	9	57%	60%	1022	1023	Normal	20%
11 Apr	11:00 AM	0, 1	9	11	61%	49%	1024	1024	Fast	60%
11 Apr	11:30 AM	0, 2	9	11	61%	49%	1024	1024	Slow	60%
1 May	07:50 PM	0, 3	5	5	86%	90%	1014	1014	Normal	100%
1 May	08:30 PM	0, 1, 2	5	5	86%	90%	1014	1014	Normal	100%
1 May	08:17 PM	0, 1, 2, 3,4, 5	5	5	86%	90%	1014	1014	Normal	100%
1 May	07:35 PM	0, 1	5	5	86%	90%	1014	1014	Normal	100%
17 May	9:00 PM	0, 1	20	21	52%	43%	1018	1020	Normal	0 %
18 May	3:00 PM	0, 1, 3	26	24	44%	55%	1014	1012	Normal	13%
19 May	1:30 PM	0, 1, 4	23	23	56%	53%	1008	1008	Normal	40%
20 May	12:30 PM	0, 1, 5	13	14	58%	49%	1022	1021	Normal	35%
21 May	9:40 PM	0, 2	14	14	71%	65%	1023	1023	Normal	0%
22 May	1:30 PM	0, 2, 3	19	21	59%	48%	1019	1018	Normal	0%
25 May	12:00 AM	0,2,4	16	17	54%	55%	1022	1023	Normal	40%
27 May	11:00 AM	0, 2, 5	20	23	55%	46%	1022	1021	Normal	0%
29 May	6:30 PM	0, 1, 2	29	29	30%	32%	1015	1015	Normal	20%
30 May	6:30 PM	0, 3	29	28	37%	36%	1014	1014	Normal	0%
31 May	6:30 PM	0, 3, 4	25	26	43%	48%	1019	1017	Normal	20%
13 Jun	3:00 PM	0, 3, 5	22	25	40%	43%	1019	1017	Normal	0%
13 Jun	7:40 PM	0, 3, 4	22	24	43%	40%	1017	1017	Normal	20%
15 Jun	5:00 PM	0, 1, 3	28	31	39%	35%	1016	1015	Normal	13%
21 Jun	5:30 PM	0, 2, 3	31	22	73%	37%	1016	1015	Normal	63%
22 Jun	5:30 PM	0, 4	31	22	73%	37%	1016	1015	Normal	63%
28 Jun	7:40 PM	0, 4, 5	23	23	60%	57%	999	999	Normal	20%
1 July	9:30 PM	0, 1, 4	19	19	60%	57%	999	999	Normal	20%
2 July	9:15 PM	0, 1, 5	17	16	77%	84%	1021	1020	Normal	40%

**Table A2:** Collected data used for evaluation of Building B. The table shows the data acquisition date, the time, the visited floors (V Floors), the indicated temperature from AccuWeather (T A) and the Weather Channel (T WC), the humidity from the same two sources (H A) and (H WC), the ambient pressure from AccuWeather (P A) and from the Weather Channel (P WC), the walking speed (W S) and the cloud coverage.

Date	Time	V Floors	T A	T WC	H A	H WC	P A	P WC	W S	Cloud Cov.
17 May	10:30 PM	0, 1	19	18	55%	45%	1018	1020	Normal	0%
18 May	9:00 AM	0, 1, 2	25	26	59%	62%	1014	1012	Normal	12%
19 May	2:00 PM	0, 1, 3	23	24	56%	53%	1008	1008	Normal	40%
20 May	1:25 PM	0, 1, 4	14	15	54%	44%	1022	1021	Normal	54%
21 May	6:00 AM	0, 1, 5	8	9	87%	88%	1026	1027	Normal	0%
22 May	2:00 PM	0, 2	19	22	59%	46%	1019	1018	Normal	59%
23 May	5:22 AM	0, 2, 3	10	11	93%	84%	1017	1018	Normal	13%
24 May	5:00 AM	0, 2, 4	17	16	67%	71%	1020	1021	Normal	20%
26 May	5:23 AM	0, 2, 5	6	8	100%	92%	1021	1021	Normal	0%
27 May	4:00 AM	0, 1, 2	24	26	43%	38%	1021	1020	Normal	0%
28 May	6:00 AM	0, 2, 3	16	17	58%	61%	1019	1019	Normal	20%
29 May	7:30 PM	0, 3	28	28	30%	32%	1014	1011	Normal	20%
30 May	5:00 AM	0, 3, 4	15	19	82%	64%	1015	1014	Normal	0%
31 May	5:00 AM	0, 3, 5	17	17	93%	94%	1019	1019	Normal	100%
12 Jun	5:00 AM	0, 1, 3	15	21	87%	63%	1015	1015	Normal	20%
13 Jun	6:00 AM	0, 2, 3	16	17	58%	61%	1019	1019	Normal	20%
19 Jun	5:00 AM	0, 4	9	13	93%	73%	1023	1022	Normal	20%
21 Jun	6:00 PM	0, 4, 5	28	31	50%	34%	1015	1015	Normal	40%
22 Jun	4:30 PM	0, 1, 4	30	31	34%	34%	1016	1015	Normal	0%
23 Jun	9:00 AM	0,2,4	25	27	60%	52%	1015	1015	Normal	90%
26 Jun	9:00 AM	0, 3, 4	21	21	64%	58%	1016	1015	Normal	0%
27 Jun	7:00 AM	0, 5	17	18	93%	93%	1012	1011	Normal	88%
28 Jun	6:00 AM	0, 1, 5	8	9	87%	88%	1026	1027	Normal	0%
30 Jun	5:00 AM	0, 1	14	13	71%	74%	1007	1007	Normal	20%
4 July	5:00 AM	0, 1	13	15	87%	77%	1022	1022	Normal	20%

**Table A3:** Collected data used for evaluation of Deutsch Akademie building. The table shows the data acquisition date, the time, the visited floors (V Floors), the indicated temperature from AccuWeather (T A) and the Weather Channel (T WC), the humidity from the same two sources (H A) and (H WC), the ambient pressure from AccuWeather (P A) and from the Weather Channel (P WC), the walking speed (W S) and the cloud coverage.

Date	Time	V Floors	T A	T WC	H A	H WC	P A	P WC	W S	Cloud Cov.
19 May	1:00 PM	0, 1	22	23	60%	56%	1008	1008	Normal	13%
22 May	10:50 AM	0, 1, 2	16	18	67%	56%	1020	1019	Normal	0%
24 May	3:30 PM	0 ,1, 3	17	17	48%	45%	1022	1022	Normal	88%
25 May	11:00 AM	0, 1, 4	16	16	54%	59%	1023	1022	Normal	40%
26 May	6:00 PM	0, 1, 5	22	23	40%	49%	1018	1018	Normal	0%
29 May	5:00 PM	0, 2	29	31	26%	30%	1015	1014	Normal	0%
30 May	6:00 PM	0, 2, 3	28	29	39%	37%	1014	1014	Normal	0%
1 Jun	7:00 PM	0, 2, 4	24	24	46%	46%	1019	1019	Normal	20%
13 Jun	3:30 PM	0, 2, 5	23	26	40%	38%	1018	1016	Normal	0%
13 Jun	7:00 PM	0, 1, 2	23	24	43%	41%	1017	1016	Normal	0%
14 Jun	9:00 AM	0, 3	16	18	67%	57%	1020	1019	Normal	0%
15 Jun	4:30 PM	0, 3, 4	27	30	67%	57%	1020	1019	Normal	0%
15 Jun	5:40 PM	0, 3, 5	28	29	41%	39%	1016	1015	Normal	13%
21 Jun	5:00 PM	0, 1, 3	31	22	73%	37%	1016	1015	Normal	63%
22 Jun	3:00 PM	0, 2, 3	30	33	34%	28%	1016	1015	Normal	13%
22 Jun	8:00 PM	0, 4	29	30	34%	28%	1016	1015	Normal	20%
23 Jun	7:30 PM	0, 4, 5	30	33	34%	28%	1016	1015	Normal	13%
26 Jun	8:30 AM	0, 1, 4	19	21	72%	61%	1016	1015	Normal	13%
28 Jun	7:00 PM	0, 2, 4	23	23	60%	56%	999	999	Normal	95%
29 Jun	2:00 PM	0, 3, 4	19	22	63%	50%	1001	1000	Normal	95%
